# Effects of endometriosis on sleep quality of women: does life style factor make a difference?

**DOI:** 10.1186/s12905-020-01036-z

**Published:** 2020-08-10

**Authors:** Samaneh Youseflu, Shahideh Jahanian Sadatmahalleh, Ghazall Roshanzadeh, Azadeh Mottaghi, Anoshirvan Kazemnejad, Ashraf Moini

**Affiliations:** 1 Department of Midwifery and Reproductive Health, Faculty of Medical Sciences, Tarbiat Modares University, Tehran, Iran; 2 Research Center for Prevention of Cardiovascular Diseases, Institute of Endocrinology & Metabolism, Iran University of Medical Sciences, Tehran, Iran; 3 Department of Biostatistics, Faculty of Medical Sciences, Tarbiat Modares University, Tehran, Iran; 4 Department of Obstetrics and Gynecology, School of Medicine, Tehran University of Medical Science, Tehran, Iran; 5 Department of Obstetrics and Gynecology, Arash Women’s Hospital, Tehran University of Medical Sciences, Tehran, Iran; 6 Department of Endocrinology and Female Infertility, Reproductive Biomedicine Research Center, Royan Institute for Reproductive Biomedicine, ACECR, Tehran, Iran

**Keywords:** Endometriosis, Sleep quality, Lifestyle, Diet, Physical activity

## Abstract

**Background:**

This study aimed to compare the lifestyle factors and SQ between women with and without endometriosis. Also in this essay, the influence of food intake, socio-demographic and clinical characteristics on sleep quality of women with endometriosis was determined.

**Methods:**

Of the 156 infertile women approached for the study, 78 women had endometriosis and 78 were included in the control group. At first, each participant completed a checklist including questions about demographics, physical activity, reproductive and menstrual status. SQ was assessed by the Pittsburgh Sleep Quality Index (PSQI). Dietary data were collected using a validated 147-item semi-quantitative FFQ.

**Results:**

Irregular menstrual status, menorrhagia, dysmenorrhea, pelvic pain, history of abortion, family history of endometriosis were associated with endometriosis risk (*P* < 0.05). In women with physical activity more than 3 h per week, high consumption of the dairy product, and fruit endometriosis is less common (*P* < 0.05). The total PSQI score, and the scores for subjective sleep quality, sleep latency, sleep disturbance domains were significantly different between the two groups (*P* < 0.05). In women with endometriosis, poor SQ was associated with dysmenorrhea, pelvic pain, dyspareunia, physical activity, and low consumption of the dairy product, fruit, and nut (*p* < 0.05).

**Conclusion:**

In endometriosis women, SQ was lower than healthy individuals. Lifestyle factors can effect on SQ of these patients.

## Background

Endometriosis is the benign proliferation of functioning endometrial glands and stroma in ectopic locations outside the uterine cavity [1], which affects an estimated range from 0.7 to 8.6% in the general population [2]. A high prevalence of dysmenorrhea, dyspareunia, non-cyclic pelvic pain, infertility and menstrual disorders (such as menorrhagia, metroragia, spotting) has been seen in these patients [1, 3]. Although the exact pathophysiology of this disease is not clear, it is believed agents that impress the volume of retrograde menstruation or affect woman’s aptitude for implantation of the endometriotic lesion are involved in the appearance and progression of endometriosis [4]. Some of the studies introduced that the appearance of endometriosis has been developed by hormonal and immunological mechanisms, genetic and inflammatory processes, as well as environmental and lifestyle factors [5].

Lifestyle factors play an important role in preventing many chronic diseases. Available evidence on the effect of lifestyle-related factors such as physical activity, nutritional status, body mass index, smoking, and SQ on endometriosis is controversial [1, 6–9]. Therefore, modified lifestyle recommendations in these patients have been discussed. For example, some studies depicted the anti-estrogenic activity of cigarette has a protective effect on endometriosis, others suggested that cigarette with an impact on pro-inflammatory gene overexpression can trigger inflammation [6, 7].

Sleep is an essential physiological process that can be impressed by many medical and sleep disorders. Several studies have demonstrated that sleep disorder is a common symptom in women with endometriosis [10, 11]. Recently, a relationship between endometriosis-related symptoms such as pain and sleep disorder has been reported [12]. Arion et al. study reveal that sleep quality of women with endometriosis is associated with poorer quality of life, more depressive symptoms, and bladder pain syndrome [13].

Sleep disorder and changes in the melatonin level can be a diverse effect on the reproductive system [14, 15]. There is compelling evidence to implicate that endogenous melatonin acts as an important analgesic, sleep inducer and regulator of the circadian rhythm, antioxidant, anticarcinogenic, immunoregulator, and anti-inflammatory agent [14, 15].

The purpose of the current study was to compare the lifestyle factors (including physical activity levels, diet, BMI, cigarette smoking), and SQ between women with and without endometriosis. Also in this essay, the influence of food intake, socio-demographic and clinical characteristics on sleep quality of women with endometriosis was determined.

## Methods

The study was conducted as a case-control study survey between May 2016 and February 2017 on 156 infertile women (78 women with endometriosis and 78 controls) that attended in Infertility Clinic of Arash Hospital in Tehran, Iran.

During the period of the study, all infertile women who undergone diagnostic laparoscopy were allocated. Based on the laparoscopy results, those women with abnormalities other than endometriosis (eg. endometrial hyperplasia, leiomyoma, and other gynecological cancer) were excluded from the study. Participants were divided into two groups: a case group consisted of 78 women with endometriosis for whom the disease was confirmed by laparoscopy and histology examinations, and a control group consisting of 78 infertile women with a normal pelvis. A control group was selected at the same time as the case group. Controls were selected randomly matched on age, education, and duration of infertility.

Eligibility criteria for inclusion in the study were including age between 18 and 45 years, absence of the history of chronic diseases or mental disorder according to self-report of women, Iranian race, Non-occurrence of bad events in the 12 months ago (e.g. death or illness of family members or close friends, financial difficulties, and etc.)

Ethical approval for the study was obtained from the Regional Ethics Committee at Tarbiat Modares University of Medical Sciences (IR.TMU.REC.1395.358). All women voluntarily participated and signed informed consent.

During the first visit, the weight and height of all women recoursing to the infertility clinic are measured. Body Mass Index (BMI) is calculated as weight divided by height squared (kg/m2).

A socio-demographic checklist including questions about socio-economic status (such as age, educational level, occupational status, income, and smoking history) was completed. The following, questions were asked about menstrual and reproductive characteristics (such as menstrual pattern, cycle regularity, menstrual duration, amount of menstrual bleeding, length of the menstrual cycle, premenstrual spotting, menarche age, presence of dysmenorrhea, dyspareunia and chronic pelvic pain, gravidity, parity, history of using contraception).

Participants were asked to report their exercise times/week. Based on their response, the participants’ physical activity was determined at three levels: low (less than 1 h per week), moderate (2–3 h per week), and high physical activity (more than 3 h per week).

A semi-quantitative 147-item FFQ was applied to obtain dietary information. This questionnaire included a list of dietary items (with standard serving measures) commonly consumed by Iranians. The Persian version of FFQ has previously been evaluated for both reliability and validity [16]. The participants were asked to report their usual food intake during the previous year on a daily, weekly, monthly and yearly basis; all these were converted to daily intakes. Portion sizes of the consumed food were transformed to grams by using domestic measurement.

All consumed food items were analyzed for their energy and nutrient components by a nutrient database (Nutritionist 4, Mosby Nutritract software, and ver.7.0, N-Squared Computing, Salem, OR, USA).

For assessment of dietary sources of melatonin, we considered food rich of tryptophan including dairy product, nut (peanut, almond, pistachio, and walnuts), fruit (banana, apple, kiwi, peach, and strawberry, orange) and meat (red meat, pultatory, and fish).

Multiple aspects of SQ during the previous month were assessed using the valid and reliable Persian version of the Pittsburgh Sleep Quality Index (PSQI) [17]. This questionnaire contains 19 items in seven categories (sleep quality, sleep latency, sleep duration, habitual sleep efficiency, sleep disturbances, use of sleeping medication, and daytime dysfunction) on a scale from 0 to 3; so the total score of PSQI is from 0 to 21. A total score higher than 5 identifies poor SQ and scores lower than 5 show the absence of sleep disorder.

Statistical analysis was performed using Statistical Package for Social Science (version 16). To check the variables’ normality, Kolmogorov–Smirnoff’s (KS) test was run. Comparisons between two groups were done using Independent Samples T-Test, Mann-Whitney’s test (MW), Chi-square tests, and logistic regression model.

## Results

Considering general risk factors, menstrual status (OR: 2.80; %95CI: 1.23–4.38; *P* = 0.01), menorrhagia (OR: 2.98; %95CI: 1.31–3.76; *P* = 0.007), dysmenorrhea (OR: 4.61; %95CI: 3.31–5.18; *P* < 0.001), pelvic pain (OR: 15.31; %95CI: 12.95–17.43; *P* < 0.001), history of abortion (OR: 2.36; %95CI: 1.05–3.28; *P* = 0.04), Family history of endometriosis (OR: 4.05; %95CI: 2.08–7.14; *P* = 0.04), and high level of physical activity (OR: 0.3; %95CI: 0.13–0.65; *P* = 0.04), were associated with endometriosis risk. There was no significant relationship between, age, age at menarche, level of education, OCP use, BMI, smoking and endometriosis risk (*P* > 0.05) (Table 1).

**Table 1:** Demographic and lifestyle characteristics of Endometriosis cases and control women

Characteristics	Case (78)	Control (77)	*P*-value	OR
Age	31.00 ± 6.63	29.35 ± 6.99	0.137	1.03(0.99–1.09)
Age at menarche				
Less than 11 year	8(10.26)	9(11.69)	0.77	0.86(0.31–2.37)
More than 12 years	70(89.74)	68(87.18)		1.00^a^
Education				
Universitically	42(53.85)	38(49.35)	0.58	0.83(0.44–1.57)
Non Universitically	36(46.15)	39(50.65)		1.00^a^
BMI	23.64 ± 4.11	23.69 ± 3.58	0.93	0.99(0.92–1.08)
OCP use				
Yes	10(13.16)	12(15.58)	0.67	0.82(0.33–1.03)
No	66(86.84)	65(84.42)		1.00^a^
Breast feeding history				
Bottle feeding	8(11.94)	5(6.49)	0.37	1.69(0.53–2.43)
Breast feeding	68(89.47)	72(93.51)		1.00^a^
Family history				
Yes	11(14.10)	3(3.90)	0.04	4.05(2.08–7.14)
No	67(85.90)	74(96.19)		1.00^a^
Abortion				
Yes	22(28.20)	11(14.29)	0.04	2.36(1.05–3.28)
No	56(71.79)	66(85.71)		1.00^a^
Menstrual status				
Regular	55(71.43)	67(87.01)	0.01	1.00^a^
Irregular	23(29.49)	10(12.99)		2.80 (1.23–4.38)
Menorrhagia				
Yes	24(30.77)	10(12.99)	0.007	2.98(1.31–3.76)
No	54(69.23)	67(87.01)		1.00^a^
Dysmenorrhea				
Yes	59(75.64)	31(40.26)	< 0.001	4.61(3.31–5.18)
No	19(24.36)	46(59.74)		1.00^a^
Pelvic pain				
Yes	44(56.41)	6(7.79)	< 0.001	15.31(12.95–17.43)
No	34(43.59)	71(92.21)		1.00^a^
Abortion				
Yes	22(28.20)	11(14.29)	0.04	2.36(1.05–3.28)
No	56(71.79)	66(85.71)		1.00^a^
Physical activity				
Less than 1 h per week	41(52.56)	26(33.77)		1.00^a^
Between 2 and 3 per week	22(28.20)	19(24.67)	0.008	0.73(0.33–1.61)
More than 3 h per week	15(19.23)	32(41.56)		0.30(0.13–0.65)
Smoking				
Yes	6(7.69)	3(3.90)	0.31	2.06(0.49–3.53)
No	72(92.31)	74(96.10)		1.00^a^

Data are demonstrated as n (%) or mean ± SD

*OR* Odds ratio, *CI* Confidence interval, *BMI* Body mass index, *OCP* Oral contraceptives

^a^Reference category

Table 2 indicates that the intake of dairy products and fruit rich in tryptophan in women with endometriosis is less than the control group (*P* = 0.02). Consumption of other food groups such as vegetables, meat, nut, fast food, and processed snacks was no significant difference between two groups (*P* > 0.05). The total PSQI score (*P* < 0.001) and the scores for the subjective sleep quality (*P* = 0.002), sleep latency (*P* < 0.001), sleep disturbance (*P* < 0.001) domains were significantly different between women with and without endometriosis.

**Table 2:** Comparison of sleep quality, and food intake between women with and without endometriosis

	Case (*N* = 78) mean ± SD	Control (*N* = 77) mean ± SD	*P*-value
Subjective sleep quality	1.35 ± 0.82	0.97 ± 0.74	0.002
Sleep latency	1.60 ± 1.07	0.96 ± 0.95	< 0.001
Duration of sleep	0.36 ± 0.68	0.32 ± 0.76	0.324
Day time function	0.97 ± 1.01	0.79 ± 0.81	0.383
Sleep disturbance	1.38 ± 0.63	0.83 ± 0.54	< 0.001
Sleep efficiency	0.47 ± 0.77	0.33 ± 0.78	0.07
Sleep medication	0.33 ± 0.81	0.23 ± 0.58	0.844
Total score	6.47 ± 3.34	4.45 ± 3.26	< 0.001
Fruit Item			
Banana	19.72 ± 17.82	24.65 ± 21.19	0.12
Peach	21.55 ± 19.30	26.72 ± 23.14	0.218
Strawberry	0.61 ± 0.71	0.83 ± 1.69	0.31
Kiwi	15.84 ± 17.59	22.85 ± 23.58	0.10
Orange	38.80 ± 28.01	51.26 ± 39.58	0.128
Apple	39.56 ± 34.13	44.22 ± 32.18	0.28
Sum	151.33 ± 78.31	188.93 ± 94.96	0.008
Dairy			
Milk	98.19 ± 104	135.45 ± 17.63	0.03
Cheese	19.23 ± 17.53	17.14 ± 13.54	0.6
Yoghurt	135.74 ± 107.04	187.13 ± 150.78	0.03
Total dairy product	317.30 ± 182.18	401.91 ± 217. 65	0.02
Nut			
Peanut	0.29 ± 0.57	0.28 ± 0.49	0.64
Pistachio	0.63 ± 0.99	0.85 ± 1.33	0.45
Walnuts	2.20 ± 3.02	2.35 ± 2.46	0.46
Almond	0.72 ± 1.24	0.66 ± 1.41	0.23
Total nut	3.85 ± 4.59	4.15 ± 4.17	0.61
Meat			
Pultatory	28.38 ± 18.16	26.81 ± 18.75	0.49
Fish	9.35 ± 8.33	12.65 ± 13.44	0.15
Red meat	20.76 ± 17.58	25.77 ± 19.70	0.07
Fast-food entrees	12.37 ± 16.09	8.87 ± 9.41	0.26
Processed snacks	12.17 ± 17.57	9.61 ± 9.72	0.37

Table 3 shows poor SQ was statistically significantly associated with dysmenorrhea (*p* = 0.03), pelvic pain (*p* = 0.02), dyspareunia (*p* = 0.04), physical activity (*p* < 0.001), and consumption of dairy product (*p* = 0.03), fruit (*p* = 0.03), and nut (*p* = 0.02).

**Table 3:** Quality of sleep among endometriosis women by socio-demographic, clinical characteristics and food intake

Variable	Good sleeper (PSQI ≥5) (*N* = 31)	Poor sleeper (PSQI < 5) (*N* = 47)	*P*-value
Age	31 ± 7.02	31.02 ± 6.31	0.99
Education			
University	17(54.84)	19(40.42)	0.21
Non university	14(45.16)	28(59.57)	
BMI	23.13 ± 3.20	23.98 ± 4.61	0.37
Mense			
Regular	26(83.87)	39(82.98)	0.92
Irregular	5(16.13)	8(17.02)	
Menorrhagia			
Yes	9(29.03)	15(31.91)	0.79
No	2270.97)	32(68.08)	
Dysmenorrhea			
Yes	20(64.52)	40(85.11)	0.03
No	11(35.48)	7(14.89)	
Dyspareunia			
Yes	11(35.48)	28(59.57)	0.04
No	20(64.52)	19(40.43)	
Pelvic pain			
Yes	12(38.71)	31(65.96)	0.02
No	19(61.29)	16(34.04)	
Physical activity			
Less than 1 h	9(29.03)	33(70.21)	
Between 2 and 3 h	10(32.26)	11(23.40)	< 0.001
More than 3 h	12(38.71)	3(6.38)	
Diet			
Dairy product	375.56 ± 157.67	278.88 ± 188.53	0.02
Red Meat	18.90 ± 16.32	21.98 ± 18.75	0.47
Fish	8.37 ± 8.39	9.99 ± 8.32	0.32
Pultatory	28.27 ± 17.38	28.45 ± 18.84	0.92
Nut	6.14 ± 7.49	2.93 ± 2.99	0.01
Fruit	227.23 ± 68.94	244 ± 75.70	0.03

## Discussion

The current study was designed to compare the SQ of women with and without endometriosis and determine the relationship between lifestyle factors and SQ in women with endometriosis. Sleep as an essential factor on the physical and mental health can be change by many agents. The main finding of the current study showed that endometriosis has a negative impact on women’s SQ. Our results also showed that scores of subjective sleep quality, sleep latency and sleep disturbance subscales were significantly less in patients than healthy women.

In consistence with our results, Leone et al.’s study showed that a high prevalence of poor SQ, daytime sleepiness, sub threshold insomnia, and moderate clinical insomnia was seen in women with endometriosis [12]. Another study demonstrated that night Shift job, especially working more than half of the shifts on a work at night, and Sleep Pattern Change on days off were associated with endometriosis risk [18]. The result of the one clinical trial demonstrates that in women with endometriosis, treatment with melatonin (10 mg for 8 weak) compared with placebo reduced daily pain, dysmenorrhea, dysuria, dyschezia, and need to use analgesic drugs, also improved SQ, and reduced the level of brain-derived neurotropic factor (BDNF) [14].

Our finding reveal that in these patient, some lifestyle factors such as exercise, diet and clinical symptoms can modify this relationship. For example, a better SQ was seen in women with endometriosis who had a diet rich in dairy products, nut, and fruits. Dairy products and some fruit (banana, kiwi, apple, peach, strawberry, and orange) are sources of tryptophan. For melatonin production, tryptophan is converted to serotonin then to N-acetyl serotonin, and finally to MLT [19]. Melatonin can regulate inflammation and immune function and protect against oxidative stress, therefore, in this way can reduce the risk of endometriosis [15]. Melatonin can also improve SQ in these women [14]*.* There is some evidence that shows sleep restriction can affect the composition of the intestinal microbiome. It seems that Mediterranean diets and other plant-rich diets with an effect on gut microbiome can improve the sleep quality of patients with cardiovascular disease [20].

It was seen that the consumption of kiwi, tart cherry juice, and oysters with the antioxidant, melatonin, and zinc contents can increase sleep duration and quality [21]. Healthy dietary patterns with low in saturated fat and high in vegetables, fruit, fiber, and seafood with potentially improving sleep quality can affect sleep [21].

Diet also considered as a main risk factor of endometriosis. Between dietary items, only consumption of the dairy product and fruit (including banana, kiwi, apple, peach, strawberry, and orange) were different between women with and without endometriosis. Some studies confirmed our results [22, 23].

Regarding the results, women with endometriosis were more likely to experience an increase in abnormal menstrual status, menorrhagia, dysmenorrhea, pelvic pain, abortion when compared with healthy women. Clinical symptoms of endometriosis such as pain and menorrhagia can worse women’s SQ. Loring et al. study demonstrated that increased deep sleep onset latency is associated with poor SQ, and besides, poor SQ leads to greater pain sensitivity the next day [10].

Our findings show that a lower level of physical activity in women with endometriosis. Regular physical exercise could be to improve levels of immune and anti-inflammatory markers and reduced menstrual flow that could lead to decreased endometriosis risk [8].On the other hand, in women with a higher level of exercise, increased levels of sex hormone-binding globulin (SHBG) can lead to reducing the level of bioavailable estrogen [8]. Insulin resistance and hyperinsulinemia have been related to endometriosis risk that physical activity can reduce these. In the result of one meta-analysis that integrated the results of nine studies, physical activity may reduce endometriosis risk, although this relationship was not statistically significant [8].

We also found that exercise is a main modifier that can improve SQ of women with endometriosis. Regular exercise can enhance melatonin secretion and in this way improve the SQ of patients with insomnia [24]. On the other hand in these women issues such as pain or abnormal cycle may decrease their tendency for exercise.

Recently, several studies were conducted on the influence of endometriosis on SQ. To our knowledge, this survey is the first research which assessed the role of life style factors on SQ of women with endometriosis. The use of validated questionnaires (eg, FFQ, and PSQI), and confirmed diagnosis through laparoscopy are other strengths of this study.

Despite the strengths of the study, some limitations should be noted. First, we don’t use a validated questionnaire for evaluating physical activity. Second, like other case-control studies, selection and recall bias are a concern for this study.

## Conclusion

Lifestyle factor such as diet and physical activity have a major impact on endometriosis risk. In women with endometriosis, SQ was lower than healthy individuals. Fruit, dairy, and nut consumption, and exercise can effect on SQ of endometriosis women. Therefore, counseling about diet and physical exercise in these women is recommended.

## Data Availability

The data sets used and analyzed during the current study are available from the corresponding author on reasonable request.

## Abbreviations

FFQ: Food Frequency Questionnaire
PSQI: Pittsburgh Sleep Quality Index
SQ: Sleep quality
SHBG: Sex hormone-binding globulin
MLT: Melatonin
